# Do glucose containing beverages play a role in thermoregulation, thermal sensation, and mood state?

**DOI:** 10.1186/1550-2783-11-24

**Published:** 2014-05-28

**Authors:** Yongsuk Seo, Corey A Peacock, John Gunstad, Keith J Burns, Brandon S Pollock, Ellen L Glickman

**Affiliations:** 1 Exercise and Environmental Physiology Laboratory, Kent State University, Kent, OH, USA; 2 Nova Southeastern University, Fort Lauderdale, FL, USA; 3 Departments of Psychology, Kent State University, Kent, OH, USA

## Abstract

**Introduction:**

Dehydration limits the appropriate delivery of oxygen and substrates to the working muscle. Further, the brain’s ability to function may also be compromised whereby thermal sensation and mood state may be altered.

**Purpose:**

The purpose of the present investigation was to compare the thermoregulatory, perceptual, and negative mood state profile in glucose (GLU) vs. non-glucose beverage (NON-GLU) condition.

**Methods:**

Ten healthy men volunteered and were counterbalanced either a GLU or NON-GLU containing beverage on separate mornings. In each condition, they were exposed to 37°C, 50% relative humidity (RH) for baseline, exercise, rehydration, and recovery periods. The exercise period elicited the desired level of dehydration (mean of 2.6 ± 0.3% body weight losses). Upon completion of the protracted exercise, participants were administered either a GLU or NON-GLU containing electrolyte based sports drink ad libitum for 30 min, followed by a recovery period of 15 min in 37°C, 50% RH. Rectal (Tre) and mean skin temperatures (Tsk) were continuously monitored. Gagge (TS) and heated thermal sensation (HTS), profile of mood state (POMS) were measure at the end of each period.

**Results:**

During recovery after rehydration, Tre was not significantly different between conditions (GLU vs. NON-GLU) (37.4 ± 0.8 vs. 37.0 ± 1.2°C); Tsk was also not affected by rehydration in both conditions (36.0 ± 0.5 vs. 36.0 ± 0.6°C) and, TS and HTS did not differ between conditions (0.9 ± 1.3 vs.1.3 ± 0.7) and (1.0 ± 0.8 vs.0.8 ± 0.3). Total mood disturbance (TMD) score for the POMS was utilized for overall negative mood state and demonstrated a main effect for time (*p* < 0.05). TMD during recovery was decreased compared to before hydration in both conditions.

**Conclusion:**

The non-glucose containing beverage maintained plasma volume and was effective at maintaining body temperature homeostasis in a similar fashion compared to the glucose containing beverage. Furthermore, negative mood state was not different between the two conditions. The non-glucose beverages can serve a valuable role in the exercise environment depending upon the sport, the ambient temperature, the individual, duration of the exercise, the age and training states of the individual.

## Introduction

Exercise in hot environments can cause a reduction in plasma volume due in part to the thermoregulation via sweating, which can decrease the blood supply to the muscle tissue. If fluid loss continues and is not replaced with water and electrolytes, body fluid distribution will then limit the appropriate delivery of oxygen and substrate to the working muscle [1]. Furthermore, heat exposure, hyperthermia, and dehydration affect the brain’s ability to function normally and can adversely impact cognitive performance whereby thermal sensation and mood state may be altered.

While much is known regarding the physiology of dehydration, the psychological effects are less clear due in part to inconsistent data in the experimental literature. Dehydration and other adverse physiological stressors have been shown to have a negative impact on mood state [2,3]. Such mood changes can then impact cognitive function [4,5].

Exercise can also impact blood glucose levels, as the body requires the use of glucose to fuel physical activity [6]. Strenuous, prolonged exercise can result in hypoglycemia, as the blood’s level of glucose may become lower because it is utilized to allow for continued physical activity. Reduced levels of glucose may exhibit as physical symptoms including shakiness, hunger, nervousness, sweating, dizziness, confusion, visual disturbance, and weakness [7]. Reduced blood sugar and the subsequent symptoms have been observed across a variety of populations following strenuous exercise, including both professional and amateur athletes [8-10]. Existing literature also indicates glucose does not directly affect hydration status. A study by Hargreaves and colleagues reported that after 40 minutes of exercise in the heat, continuous administration of glucose did not alter plasma volume or hydration status [11]. These results may be attributes to experimental methodologies, such as timing of fluid replacement and environmental conditions (i.e., temperature and exercise duration), both of which can impact fluid homeostasis.

Additionally, it has been accepted by the American College of Sports Medicine that it is appropriate to consume a drink that is comprised of glucose and electrolytes to combat the loss of these ingredients during exercise and the stressor of heat [12]. Many sport beverages contain glucose and additional nutritional components, specifically electrolytes (i.e., sodium, potassium, vitamin B12, etc.) which element(s) benefitted cognitive function relative to water. Therefore, rehydration with comparable beverages, with the exception of carbohydrate content, would allow for more accurate examination of between-condition differences.

The purpose of the current investigation is to examine the effects of a fluid replacement drink that contains electrolytes, glucose and calories versus a fluid replacement drink containing solely electrolytes (GLU), non-digestible artificial sweeteners, and zero calories (NON-GLU) on rectal temperature, skin temperature, and mood state after protracted exercise in 37°C for 90 minutes. It was hypothesized that a GLU containing drink will elicit improved mood state during recovery after prolonged exercise in the heat compared to a NON-GLU beverage. The findings increase our knowledge and safety for exercise in the heat and the role of glucose on mental and physiological processes during rehydration.

## Methods

### Subjects

Ten males (22 ± 2 yrs, 181.4 ± 6.6 cm, 88.4 ± 10.4 kg) volunteered to take part in the current investigation and reported to the laboratory on three occasions (preliminary, GLU, NON-GLU). Through completion of a medical history screening, subjects were excluded with the presence or history of medical, neurological, developmental, or psychiatric disorders or a history of heat illness. The sample consisted of males, as exercise intensity and duration could be confounded with a co-ed sample (i.e., males vs. females may require a different level of exercise to produce the level of dehydration desired) [13-15]. Further, only Caucasian males were utilized, as non-whites and female have demonstrated differences in thermoregulation [16,17]. The study protocol was approved by the Institutional Review Board at Kent State University. All subjects provided written informed consent before participating.

### Measurements

Rectal temperature (Tre) was measured by a thermistor inserted 13 cm into the rectum (ER400-12, Respiratory Diagnostic Products, Irvine, CA). Skin thermistors (Model 409B, Yellow Springs, OH) were used to measure skin temperature at the following sites: chest, triceps, forearm, thigh, and calf [18]. Rectal, skin and air temperatures were collected by an interface (iNet-100HC, Omega Engineering, Stamford, CT). Mean skin temperature (Tsk) was calculated using the formula supported in the current literature [18]: Tsk = (0.22 × calf temperature) + (0.28 × thigh temperature) + (0.28 × chest temperature) + (0.14 × forearm temperature) + (0.08 × triceps temperature).

Participants’ metabolic rate (VO_2_) was assessed via Parvo metabolic measurement system (Parvo Metabolic Cart, Sandy, Utah), which analyzed expired air samples via an indirect automated open circuit system to determine oxygen consumption.

The Profile of Mood State-Short Form (POMS-SF) is a 37 item, condensed version of the original Profile of Mood State questionnaire which preserves the six measures of mood disturbance. The questionnaire consists of a five-point Likert scale, with mood-related items that provide answers ranging from 0 (not at all) to 4 (extremely) to answer the question, “how are you feeling right now?”. The POMS-SF yields six subscales including fatigue-inertia, vigor-activity, tension-anxiety, depression-dejection, anger-hostility, and confusion-bewilderment.

The thermal sensation was measured using the Gagge thermal sensation scale (TS) [19] and heated thermal sensation (HTS) [20], both of which are valid and reliable measures of subjective whole body thermal sensation. Participants were asked to quantify their thermal sensation utilizing these scales.

### Procedures

**Figure 1:**
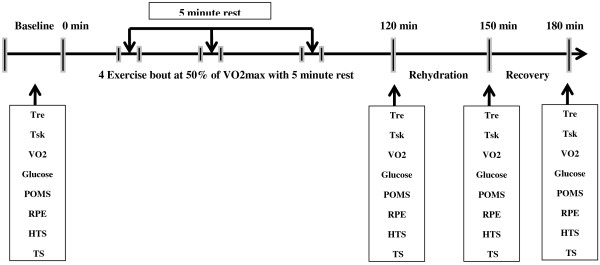
The experimental procedure and time line.

During the initial visit, in order to determine cardiovascular fitness and capacity, resting and peak blood pressure, resting and peak heart rate (HR), and peak oxygen uptake (VO_2_max) data were collected. The graded exercise test (GXT) was conducted on an electronically braked cycle ergometer (Lode, Quinton Excalibur, Netherlands). The expired air was analyzed for oxygen and carbon dioxide concentration using an automated open circuit system to determine maximal oxygen consumption (VO_2_max). Following completion of the VO_2_max test and health history questionnaire, those participants deemed eligible for participation were then scheduled for two additional counterbalanced (GLU and NON-GLU) testing sessions. All experiments were conducted in the morning hours following an overnight fast. Each counterbalanced experimental trial (GLU vs. NON-GLU), lasted approximately 180 minutes (Figure 1). Prior to the experimental trials, participants were provided a standardized breakfast (a bagel and a banana) and water (500 ml) intake to minimize possible confounds. During each experimental trial (GLU vs. NON-GLU), a baseline measure of Tre, Tsk, VO_2_, profile of mood state, thermal sensation [19] and Heated thermal sensation [20] were collected in an environmentally-controlled room set at 37°C and 50% RH. Participants were then asked to exercise on a cycle ergometer in the climatically controlled chamber, inducing an average dehydration of 2.6 ± 0.3% of their initial body weight. In order to assess the individuals percentage of body weight lost, they were asked during this period to exercise for 25-minute intervals, with interspersed 5 minutes rest periods to measure weight loss. Cycling intensity was set to 50% of the participants VO_2_max. Prior to the completion of every exercise bout, during minutes 22–25, data was collected for thermal sensation, metabolic rate, Tre, and Tsk. The individuals were then weighed during the 5 minute rest period.

These 25-minute exercise blocks and 5-minute assessment blocks were continued until desired level of dehydration of loss of 2.6% of body weight or a maximum of 4 exercise bouts (total of 100 minutes of exercise) were achieved.

Immediately following the last exercise protocol, and still in the 37°C chamber, participants were assessed for TS, VO_2_, Tsk, POMS and Tre. Following these assessments, participants received a fluid replacement drink consisting of GLU or NON-GLU. They were permitted to drink ad-libitum for 30-minutes to allow for adequate re-hydration. The quantity consumed by each participant was recorded.

Tre, Tsk, VO_2_, POMS, and thermal sensation data were recorded for 30 minutes after the rehydration period.

### Statistical analyses

Using SPSS 17.0, two-way repeated measures analyses of variance (condition and time) were performed for Tre, Tsk, VO_2_, POMS, TS, and HTS. The level of significance was set a priori at *p* ≤ 0.05 and to examine the main effects of time; the dehydrated state (immediately post last exercise bout) and most rehydrated (immediately post rehydration bout) and condition (GLU vs. NON-GLU). If a significant interaction was found, post-hoc paired sample t-tests were utilized. The POMS was administrated four total times per trial. However, the main goal of this study was on the post-rehydration recovery mood state.

## Results

The amount of fluid consumed during the rehydration periods was not statistically different from one another (*p = 0.997)* with an average of 987.5 ± 197.3 ml consumed via the GLU replacement drink and 990.0 ± 224.1 ml consumed via the NON-GLU replacement drink. Therefore, any difference in physiological measures detected between conditions is not a result of differing amounts of re-hydration drinks consumed.

Baseline measures of the Baseline measures of Tre, Tsk, VO_2_, POMS, TS, and HTS and were assessed within 10 minutes upon entering an environmentally controlled chamber set 37°C. Baseline physiological measurements were similar between conditions. In particular, Tre (37.3 ± 0.3 vs. 37.0 ± 0.5°C) and Tsk (34.7 ± 1.4 vs. 35.1 ± 0.5°C), Glucose level (115.3 ± 19.6 vs. 127.1 ± 23.1 ml/dl), and VO_2_ (4.9 ± 1.3 vs. 5.5 ± 2.7 ml/kg/min) were not different between GLU and NON-GLU, respectively. In addition, baseline POMS TMD (−2.8 ± 11.1 vs. -4.3 ± 8.5), TS (1.5 ± 0.7 vs. 1.5 ± 0.7), and HTS (1.4 ± 1.4 vs. 0.9 ± 0.5) were not different between two conditions, respectively. After dehydration (2.6% of body weight loss) Tre and Tsk were elevated in both conditions (Table 1). However, there were no significant differences in Tre and Tsk between conditions. Despite the elevated body temperature, metabolic rate did not increase compared to baseline and no difference was found between two conditions. The blood glucose was decreased compared to baseline but there was no significant difference observed between groups. These data showed that upon completion of the exercise bout both conditions were equally dehydrated and in similar physiologic states. POMS TMD at dehydration increased compared to baseline values, but no difference was exhibited between conditions.

**Table 1:** Physiological and thermal sensation response to heat exposure

	**Baseline**		**Dehydration**		**Rehydration**		**Recovery**	
	**GLU**	**NON-GLU**	**GLU**	**NON-GLU**	**GLU**	**NON-GLU**	**GLU**	**NON-GLU**
Tre	37.3 ± 0.3	37.0 ± 0.5	37.8 ± 1.2	37.9 ± 0.5	37.7 ± 0.8	37.7 ± 0.5	37.4 ± 0.8	37.0 ± 1.2
Tsk	35.2 ± 0.5	37.0 ± 0.5	36.5 ± 0.5	36.0 ± 1.2	35.0 ± 0.6	36.5 ± 0.6	36.0 ± 0.5	36.0 ± 0.6
VO_2_	4.9 ± 1.3	5.5 ± 2.7	4.9 ± 1.5	4.4 ± 0.8	4.9 ± 1.1*	4.2 ± 0.7	5.5 ± 1.0*	4.3 ± 1.2
TS	1.5 ± 0.7	1.5 ± 0.7	2 ± 1.0	1.8 ± 0.9	1.3 ± 0.8	0.9 ± 0.6	0.9 ± 1.3	1.3 ± 0.7
HTS	1.4 ± 1.4	0.9 ± 0.5	2.9 ± 2.5	1.7 ± 1.4	1.4 ± 1.2	0.9 ± 1.2	1.0 ± 0.8	0.8 ± 0.3

Data are Mean ±SD. *denotes significant difference from NON-GLU condition at same time (*p* < 0.05).

Rectal temperature (Tre), mean skin temperature (Tsk), metabolic rate (VO_2_), Gagge (TS) and heated thermal sensation (HTS).

Upon completion of the rehydration period, there was no significant difference between conditions for Tre and Tsk. Expectedly, metabolic rate was different between conditions after rehydration. An average the VO_2_ of 4.9 ± 1.1 ml/kg/min observed in the glucose electrolyte containing beverage and the average VO_2_ 4.3 ± 1.2 ml/kg/min observed in the non-glucose electrolyte containing beverage (*p* = 0.007). In addition, blood glucose levels in GLU condition statistically greater compared to NON-GLU fluid replacement drink were (*p = 0.000).* However, in both thermal scales, there is no significantly different between two conditions.

**Figure 2:**
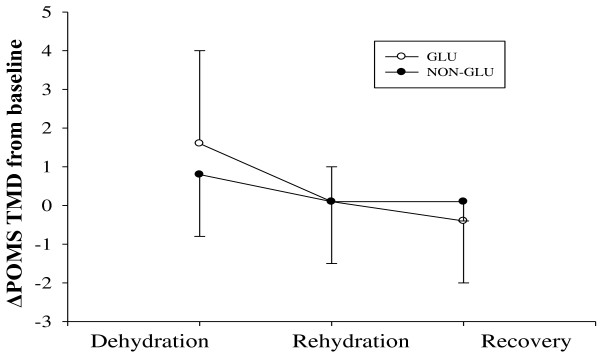
Delta POMS-SF total score with higher scores indicative of greater mood-related symptoms and thus poorer mood.

Following the recovery period, there was no significant difference between the two conditions on Tre, Tsk, and both thermal scale. However, oxygen consumption was significantly higher in GLU condition compared to NON-GLU condition. Furthermore, blood glucose level remained higher in GLU condition compare to NON-GLU condition (*p* = 0.009). The change in POMS TMD demonstrated no main effect for condition (*p* = 0.554), time (*p* = 0.273), and time by condition interaction (*p* = 0.053). Analyses of paired sample *t*-test showed that POMS TMD was decreased compared to before rehydration. However, did not differ between conditions (GLU vs. NON-GLU) (Figure 2).

## Discussion

The purpose of this study was to quantify changes in mood state during and following intake of fluid in hot ambient condition. The results of this study elucidate the need for fluid during exercise in the heat; however, the fluid does not need to contain high glucose or calories to maintain homeostasis.

With the amount of calories that individuals consumes daily, and the amount of ergogenic aids marketed for post-exercise rehydration the data presented is noteworthy. For the most part, investigators believe a high caloric type of beverage is the optimal hydration beverage during prolonged exercise in the heat and the subsequent recovery process. Results from the current study contradict this theory. Although, glucose is utilized during strenuous exercise, it is the loss of electrolytes via sweat that contributes mostly to the hypohydration of athletes [21]. As indicated by the statistical analyses provided, there were no differences in amount of liquid consumed after the strenuous exercise bout in the heat between the GLU and NON-GLU conditions. Additionally, rectal and skin temperature also demonstrated that there are no significant differences between conditions. This provides support that the main mechanism of controlling body temperature is not mediated by glucose, simply due to the consumption of liquid and electrolytes. However, significant differences were indicated between the conditions in subsequently metabolic rate. The VO_2_ is directly associated with the full-calorie drink (i.e., ≈ 220 calories/960 ml). VO_2_ is significantly higher due to the thermic effect of feeding, whereas the higher blood glucose is attributed to the sugar (56 g of sugar/960 ml) in the full-calorie drink, or, ≈ 220 calories. These two variables being significantly higher will to lead to an inhibition of fat metabolism. Inhibiting fat metabolism is detrimental reducing body fat and consequently is one of the many factors that contribute to obesity [22].

Additionally, the increased metabolic rate observed in the full-caloric condition could have an impact on exercise recovery and subsequent exercise bouts. No differences were observed between rectal and skin temperature between conditions at the conclusion of the post re-hydration period indicating a similar level of recovery and thermal homeostasis were achieved between the differing fluid replacement drinks. However, due to the thermic effect of food and the energy needed for the active process of carbohydrate absorption and subsequent breakdown and utilization the increased metabolic rate observed in the full-calorie condition may have an impact on long term exercise recovery [22]. Instead of the recovery and rebuilding of muscle damaged during the exercise bouts, the body is using additional energy and physiologic processes to aid in the digestion of the glucose absorbed. Further investigation is needed to determine the long term recovery and exercise performance between a full calorie and eucaloric fluid replacement drink.

The eucaloric drink was equally effective in maintaining temperature homeostasis, thus rejecting the hypothesis of the researchers. Although no significant differences were detected between the volume of fluid replacement drink consumed, subjects did drink slightly more of the eucaloric beverage. This small increased consumption of the eucaloric beverage in the 30-min period post exercise may support evidence that the high glucose containing beverages are less palatable than non-glucose containing beverages. Davis and colleagues reported that subjects after exercise in heat drank less of a high glucose drink due to the onset of nausea [23]. More research is needed in this area to provide further evidence of the palatability of glucose containing beverages. Furthermore, since the sodium is present in the NON-GLU drink it was equally effective in maintaining plasma volume more so than a water alone beverage [21].

Some limitations could be identified in the present study. Dehydration state was confirmed by weight loss and change of Tre (0.7°C). However, it would be beneficial to include other assessments of hydration status such as urine specific gravity or plasma osmolality. Although urine specific gravity or plasma osmolality are widely used to determine dehydration status in research and clinical setting [24], these techniques were not used during this study. Thus, we were not able to directly determine the effect of dehydration state on mood state. Other limitations include studying only the physically active young population and testing a single aspect of mood state. Hence, a wide range of subjects (e.g., women and older population) and additional measurements of mood state will be needed for future experiments.

## Conclusion

The non-glucose containing beverage maintained plasma volume and was effective at maintaining body temperature homeostasis in a similar fashion compared to the glucose containing beverage. Furthermore, negative mood state was not different between the two conditions. The non-glucose beverages can serve a valuable role in the exercise environment depending upon the sport, the ambient temperature, the individual, duration of the exercise, the age and training states of the individual.

## Competing interests

The authors declare that they have no competing interests.

## Authors’ contributions

All authors contributed equally to this manuscript. All authors have read and approved the final manuscript.

## References

[B1] Sawka MN Montain SJ Fluid and electrolyte supplementation for exercise heat stress Am J Clin Nutr 2000 72 564S 572S 10919961 10.1093/ajcn/72.2.564S

[B2] D’Anci KE Vibhakar A Kanter JH Mahoney CR Taylor HA Voluntary dehydration and cognitive performance in trained college athletes Percept Mot Skills 2009 109 1 251 269 10.2466/pms.109.1.251-269 19831106

[B3] Choma CW Sforzo GA Keller BA Impact of rapid weight loss on cognitive function in collegiate wrestlers Med Sci Sports Eexerc 1998 30 5 746 749 10.1097/00005768-199805000-00016 9588618

[B4] Herrmann LL Le Masurier M Ebmeier KP White matter hyperintensities in late life depression: a systematic review J Neurol Neurosurg Psychiatry 2008 79 6 619 624 17717021 10.1136/jnnp.2007.124651

[B5] Nebes RD Pollock BG Houck PR Butters MA Mulsant BH Zmuda MD Reynolds CF 3rd Persistence of cognitive impairment in geriatric patients following antidepressant treatment: a randomized, double-blind clinical trial with nortriptyline and paroxetine J Psychiatr Res 2003 37 2 99 108 10.1016/S0022-3956(02)00085-7 12842163

[B6] McMahon SK Ferreira LD Ratnam N Davey RJ Youngs LM Davis EA Fournier PA Jones TW Glucose requirements to maintain euglycemia after moderate-intensity afternoon exercise in adolescents with type 1 diabetes are increased in a biphasic manner J Clin Endocrinol Metab 2007 92 3 963 968 10.1210/jc.2006-2263 17118993

[B7] Cryer PE Symptoms of hypoglycemia, thresholds for their occurrence, and hypoglycemia unawareness Endocrinol Metab Clin North Am 1999 29 3 495 500 v-vi 10500927 10.1016/s0889-8529(05)70084-0

[B8] Billat VL Demarle A Slawinski J Paiva M Koralsztein JP Physical and training characteristics of top-class marathon runners Med Sci Sports Exerc 2001 33 12 2089 2097 10.1097/00005768-200112000-00018 11740304

[B9] di Prampero PE Atchou G Bruckner JC Moia C The energetics of endurance running Eur J Appl Physiol Occup Physiol 1986 55 3 259 266 10.1007/BF02343797 3732253

[B10] Rapoport BI Metabolic factors limiting performance in marathon runners PLoS Comput Biol 2010 6 10 1 13 10.1371/journal.pcbi.1000960 PMC2958805 20975938

[B11] Hargreaves M Angus D Howlett K Conus NM Febbraio M Effect of heat stress on glucose kinetics during exercise J Appl Physiol 1996 81 4 1594 1597 8904574 10.1152/jappl.1996.81.4.1594

[B12] Sawka MN Burke LM Eichner ER Maughan RJ Montain SJ Stachenfeld NS American College of Sports M American College of Sports Medicine position stand. Exercise and fluid replacement Med Sci Sports Exerc 2007 39 2 377 390 10.1249/mss.0b013e31802ca597 17277604

[B13] Bar-Or O Effects of age and gender on sweating pattern during exercise Int J Sports Med 1998 19 Suppl 2 S106 S107 9694411 10.1055/s-2007-971970

[B14] Kelley GA Lowing L Kelley K Gender differences in the aerobic fitness levels of young African-American adults J Natl Med Assoc 1999 91 7 384 388 10643210 PMC2608467

[B15] Mehnert P Brode P Griefahn B Gender-related difference in sweat loss and its impact on exposure limits to heat stress Int J Ind Ergonom 2002 29 6 343 351 10.1016/S0169-8141(02)00073-2

[B16] Kaciuba-Uscilko H Grucza R Gender differences in thermoregulation Curr Opin Clin Nutr Metab Care 2001 4 6 533 536 10.1097/00075197-200111000-00012 11706289

[B17] Hessemer V Bruck K Influence of menstrual-cycle on shivering, skin blood-flow, and sweating responses measured at night J Appl Physiol 1985 59 6 1902 1910 4077797 10.1152/jappl.1985.59.6.1902

[B18] Toner MM Sawka MN Foley ME Pandolf KB Effects of body mass and morphology on thermal responses in water J Appl Physiol 1986 60 2 521 525 10.1063/1.337441 3949658

[B19] Gagge AP Stolwijk JAJ Hardy JD Comfort and thermal sensations and associated physiological responses at various ambient temperatures Environ Res 1967 1 1 1 20 10.1016/0013-9351(67)90002-3 5614624

[B20] Glickman EL Peacock C Gunstad J Kakos L Burns KJ Pollock B Feeback M Seo Y A thermal perception scale for use during rest and exercise in 37°C ambient air [abstract] Med Sci Sports Exerc 2013 45 5 S70

[B21] Armstrong LE Exertional Heat Illnesses 2003 Champaign, IL: Human Kinetics

[B22] Brooks GA Fahey TD Baldwin KM Exercise Physiology: Human Bioenergetics and Its Applications with PowerWeb Bind-in Card 2004 New York, NY: McGraw-Hill Higher Education

[B23] Davis JM Burgess WA Slentz CA Bartoli WP Pate RR Effects of ingesting 6% and 12% glucose/electrolyte beverages during prolonged intermittent cycling in the heat Eur J Appl Physiol Occup Physiol 1988 57 5 563 569 10.1007/BF00418463 3396573

[B24] Armstrong LE Assessing hydration status: the elusive gold standard J Am Coll Nutr 2007 26 Suppl 5 575S 584S 17921468 10.1080/07315724.2007.10719661

